# Supplementary material for: Pork Meat Composition and Health: A Review of the Evidence
Source: Foods. 2024 Jun 17;13(12):1905. doi: 10.3390/foods13121905 (PMC11202857; doi:10.3390/foods13121905)
Supplement: Supplementary file 1 [file foods-13-01905-s001.zip › foods-3034665-supplementary.pdf]

**Supplementary material**

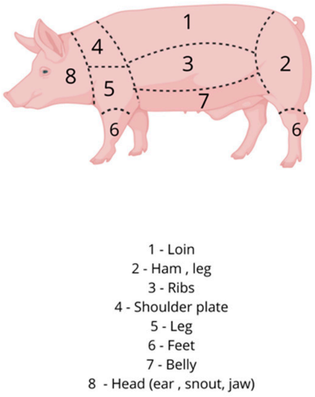


**Figure S1 –** Pork meat cuts diagram
